# Mortality, hospital days and expenditures attributable to ambient air pollution from particulate matter in Israel

**DOI:** 10.1186/s13584-016-0110-7

**Published:** 2016-11-15

**Authors:** Gary M. Ginsberg, Ehud Kaliner, Itamar Grotto

**Affiliations:** Israel Ministry of Health, Public Health Services, Yirmiahu Street 39, Jerusalem, 9446724 Israel

**Keywords:** Attributable mortality, Hospitalisations, Air pollution, Particulate matter

## Abstract

**Background:**

Worldwide, ambient air pollution accounts for around 3.7 million deaths annually. Measuring the burden of disease is important not just for advocacy but also is a first step towards carrying out a full cost-utility analysis in order to prioritise technological interventions that are available to reduce air pollution (and subsequent morbidity and mortality) from industrial, power generating and vehicular sources.

**Methods:**

We calculated the average national exposure to particulate matter particles less than 2.5 μm (PM2.5) in diameter by weighting readings from 52 (non-roadside) monitoring stations by the population of the catchment area around the station. The PM2.5 exposure level was then multiplied by the gender and cause specific (Acute Lower Respiratory Infections, Asthma, Circulatory Diseases, Coronary Heart Failure, Chronic Obstructive Pulmonary Disease, Diabetes, Ischemic Heart Disease, Lung Cancer, Low Birth Weight, Respiratory Diseases and Stroke) relative risks and the national age, cause and gender specific mortality (and hospital utilisation which included neuro-degenerative disorders) rates to arrive at the estimated mortality and hospital days attributable to ambient PM2.5 pollution in Israel in 2015. We utilised a WHO spread-sheet model, which was expanded to include relative risks (based on more recent meta-analyses) of sub-sets of other diagnoses in two additional models.

**Results:**

Mortality estimates from the three models were 1609, 1908 and 2253 respectively in addition to 184,000, 348,000 and 542,000 days hospitalisation in general hospitals. Total costs from PM2.5 pollution (including premature burial costs) amounted to $544 million, $1030 million and $1749 million respectively (or 0.18 %, 0.35 % and 0.59 % of GNP).

**Conclusions:**

Subject to the caveat that our estimates were based on a limited number of non-randomly sited stations exposure data. The mortality, morbidity and monetary burden of disease attributable to air pollution from particulate matter in Israel is of sufficient magnitude to warrant the consideration of and prioritisation of technological interventions that are available to reduce air pollution from industrial, power generating and vehicular sources. The accuracy of our burden estimates would be improved if more precise estimates of population exposure were to become available in the future.

**Electronic supplementary material:**

The online version of this article (doi:10.1186/s13584-016-0110-7) contains supplementary material, which is available to authorized users.

## Background

According to the WHO, air pollution accounted in 2012 for around 7,000,000 deaths worldwide [1], of which 3,700,000 deaths were attributable to ambient air pollution (AAP) as opposed to household air pollution [2]. The major contributor to AAP is ambient particulate matter pollution (APMP), with ambient ozone pollution being a minor contributor [2]. In 2005 and 2010, it was estimated that there were around 565,000 and 500,000 deaths respectively in the WHO European region attributable to APMP, of which 2552 and 2452 deaths respectively occurred in Israel [1].

The WHO mortality calculations were primarily made by multiplying average pollution levels by cause specific relative risks (RR) based on the literature [3–6]. An unpublished study commissioned by the Israeli Ministry of Environment protection [7], based on aggregation of spatial emission rates from all pollutants, estimated the monetary costs of air pollution from transport, industrial and electricity generation sources, but did not estimate mortality.

Measuring the burden of disease from air pollution is important not just for advocacy but also is a first step towards carrying out a full cost-utility analysis in order to prioritise technological interventions that are available to reduce air pollution (and subsequent morbidity and mortality) from industrial, electricity generating and vehicular sources.

This paper aims to estimate mortality, serious morbidity (proxied by hospitalization days) and associated expenditures from APMP in Israel.

## Methods

### Population-weighted PM2.5 exposure

Annual average ambient PM2.5 and/or PM10 exposure data was calculated based on published monthly data for 2015 from 52 non-roadside monitoring stations [8]. Readings from stations that only recorded PM10 were converted to PM2.5 by a monthly specific PM2.5/PM10 ratio based on stations where both measurements were made in the same region or on national data in the event no regional data existed.

Mid-2015 population data by towns, cities and regions (by urban and rural status) were multiplied by the relevant local monitoring stations annual average PM2.5 level and divided by the national exposed population figure of 8,608,500 (which included 236,000 temporary migrants) in order to arrive at the national population weighted average PM2.5 exposure level [9, 10].

Where more than one monitoring station existed in a city, an average PM2.5 value was calculated and applied to that city’s population. Separately weighted urban and rural regional average readings for each geographic region were calculated and applied to other urban and rural populations which were not covered by a monitoring station.

### Relative risks

Age group (in five year increments) specific RR, based on the WHO burden of disease calculations from AAP [11], were obtained for ischemic heart disease (IHD) and cerebrovascular disease (stroke) mortality from PM2.5 in adults aged over 25 years. Non-age specific RR were obtained for chronic obstructive pulmonary disease (COPD), lung cancer (LC) as well as for acute lower respiratory infection (ALRI) in children under 5 years of age. We utilised a test version of a spread-sheet for estimating the burden of disease from ambient air pollution that we obtained from the WHO (based on the methods described in http://www.who.int/phe/health_topics/outdoorair/databases/AAP_BoD_methods_March2014.pdf?ua=1 and http://www.who.int/phe/health_topics/outdoorair/databases/en/). Values reported in terms of PM10 were converted to PM2.5 equivalents by multiplying by 0.73 [12].

### Sensitivity analyses (Table 1)

**Table 1:** Diagnostic composition of different models (ages 25+ unless otherwise stated)

	ICD10 code	WHO	MAXI^a^	WIDE^a^
Ling Cancer	C33-C34	X	X	X
Diabetes Type II	E11		X	X
Dementia	F01–F07			X^b^
Parkinson’s	G20			X^b^
Alzheimer’s	G30			X^b^
Circulatory	I10–I99			X
Cardiovascular	I20–I28, I30–I52			
	I60–I79			
IHD	I20–I25	X	X	
CHF	I30–I52		X	
Stroke	I60–I61, I63, I64	X	X	
	I69.0–I69.1			
Respiratory	J00–J99			X
ALRI	J10–J22	X^c^	X^d^	
COPD	J44	X	X	
Asthma	J45			X
Low Birth Weight	P07.0–P07.1		X^e^	X^e^

^a^Based on literature up to and including 2015

^b^Only for hospitalization days not for mortality

^c^Under 1 year old only

^d^All ages

^e^Under five years old only

The WHO supplied RR values were only based on literature that was available up to mid-2013. We updated these RR by including recent papers and meta-analyses of incidence, utilization and mortality data and expanded the categories in the test tool model to include type 2 Diabetes in Adults [13] and Asthma [14, 15] and Low Birth Weight [LBW] in the under-fives [16] in what we call our MAXI (category) model.

A recent study of 9.8 million subjects in the USA [17] reported that PM2.5 levels were positively related to elevated hospitalization risks for Alzheimer’s disease, Parkinson’s disease and dementia. The results indicated that long-term changes in PM2.5 accelerated neuro-degeneration, potentially after the disease onset, hence we included the attributable hospitalization days into our MAXI model. However, we did not include estimates of attributable mortality, since the study was unable to assess whether PM2.5 levels caused the onset of neuro-degeneration, for which age is a predominant risk factor [18].

We applied age-specific relative risks for IHD and stroke in proportion to the overall ratio of the RR calculated from the meta-analyses to the overall RR from the WHO model.

We noticed that different meta-analyses of the long-term effect (short-term effects were excluded) of pollutants on a specific disease, did not always include identical studies. Due to time constraints, in our calculation of updated relative risks, we included every individual study that had been included in meta-analyses, plus any published data since the latest meta-analysis. However we took care not to include multiple studies based on the same temporal populations and preserving a hierarchy of inclusion based primarily on mortality, then hospitalisations, emergency room visits and incidence risks (which we assumed will reflect proportionality of pollution related risks).

However, we excluded studies based in the Far East (China, South Korea, Japan etc.) as their risks (which were usually higher) were generally based on higher levels of air pollution than that of Israel, North America and Europe [19].

In addition we included a WIDE category model, that included the broad areas of all circulatory and all respiratory diseases in addition to lung cancer, diabetes and LBW.

Combined RR were calculated by applying weights inversely proportional to the square of the reported standard errors of the estimates of the diseases in the WIDE and MAXI categories.

### Population Attributable Fraction (PAF)

Age, gender and cause specific PAFs for APMP were calculated according to the standard formula

$$ \mathrm{P}\mathrm{A}\mathrm{F}=\frac{\mathrm{RR}-1}{\left(\mathrm{R}\mathrm{R}-1\right)+1} $$

### Attributable mortality and hospital days

Age and cause specific mortality and days of hospital utilisation by primary cause of death and hospitalisation for 2009–2013 were obtained from the Ministry of Health’s national mortality and hospitalisation data bases. These raw data were adjusted upwards by 6.8 % [9] to take into account population growth until mid-2015. Finally we calculated mortality and hospital days attributable to PM2.5 by multiplying the age, gender and cause specific mortality and hospitalization data by the relevant PAF.

### Potential years of Life Lost (PYLL)

Extrapolations of age and gender specific life expectancies to 2015 [10, 11] were multiplied by age-gender and cause specific mortality data in order to calculate the cause specific PYLL attributable to PM2.5.

### Disability adjusted life years (DALYs) lost

Age- and gender-specific disability weights, used by the Ministry of Health, were applied to the life expectancies in order to calculate each individual’s additional Healthy Adjusted Life Expectancy (HALE), using a 3 % per annum discount rate. These HALEs were subsequently multiplied by age-gender and cause specific mortality data in order to calculate the cause specific DALYs lost due to mortality.

### Attributable direct costs of ambient PM2.5 pollution

In 2015, Israel spent around $18.5 billion on health services [9, 10]. Around 57 % of this was spent on capital costs, medicines, equipment and ambulatory, emergency room and out-patient visits [9, 10]. This figure was in turn multiplied by the percentage of hospital days from APMP for each of our models. The general hospitalisation costs (accounting for a further 19.6 %) were then added, taking into account that the per diem hospital costs were higher in departments [$916 vs $869] that cared for persons with diagnoses affected by PM2.5 than the average hospital cost [20].

We included premature burial costs (based on discounting the $5263 average burial costs over the life years lost) as the only monetary cost (in contrast to “human costs” reflected in lost DALYs) attributable to mortality. In addition, we calculated a statistical value of life loss based on valuing each member of society [regardless of age and gender] according to the national average gross national product (GNP) per capita of $35,222 multiplied by their life expectancy, using a 3 % per annum discount rate.

The hospital, health service and premature burial costs were also expressed in terms of their percentages of GNP. However since the statistical value of life computation is based on “virtual” as opposed to real resource costs, this was not expressed in terms of percentage of GNP.

## Results

The population weighted average PM2.5 exposure in Israel in 2015 was 21.6 μg/m^3^. The calculated diagnostic specific RR due to 10 μg/m^3^ changes in PM2.5 that we used for the non-WHO models are listed along with their diagnoses in Additional file 1: Appendix I. Risks for ALRI (RR = 1.10, 95 % CI 1.06–1.12), Alzheimers (3.00, 2.40–3.70), Asthma (1.02, 1.01–1.03), Dementia (1.16, 1.10–1.22), Diabetes (1.05, 1.01–1,08), IHD (1.11, 1.08–1.15), Lung Cancer (1.11, 1.05–1.16), Parkinson’s (1.88, 1.44–2.40) and Respiratory Diagnoses (1.04, 1.001–1.08) were all significant. COPD (1.03, 0.997–1.07) and LBW (1.06, 0.989–1.12) were marginally not-significant, whilst there was a non-significant elevated risk for Strokes [1.08, 0.93–1.24].

According to the WHO model, 1609 (95 % CI 863–2361) deaths (or 3.6 % of all fatalities) were attributable to ambient PM2.5. Around half were due to IHD and a quarter attributable to strokes (Table 2).

**Table 2:** Mortality attributable to ambient air pollution from PM2.5 (Israel 2015) *(WHO model)*

	Deaths	95 % Lcl	95 % Ucl	Discounted		
				PYLL	HALE losses	HALE losses
Lung cancer	232	55	380	3952	3056	2249
COPD	110	44	189	1178	866	695
IHD	850	586	1112	10,460	7958	6075
Stroke	417	175	680	4937	3678	2842
ALRI	1	0	1	42	36	15
TOTAL	1609	861	2361	20,569	15,595	11,877

The Wide list (containing wide circulatory and respiratory categorisations) estimated 15 % more deaths (1908, 95 % CI 1121–2804 being 4.3 % of all deaths) than the WHO model, Circulatory disorders accounted for 64 % of attributable mortality, with lung cancer and respiratory disorders each accounting for 18 % and 14 % respectively (Table 3).

**Table 3:** Mortality attributable to ambient air pollution by pollutant (Israel 2015) *(WIDE list)*

PM2.5	Deaths	95 % Lcl	95 % Ucl	Discounted		
				PYLL	HALE losses	HALE losses
Diabetes	78	24	122	820	600	481
LBW	0	−0	0	17	15	6
Lung cancer	349	199	478	5945	4598	3384
Circulatory	1217	890	1585	12,445	9181	7274
Respiratory	265	8	618	2776	2047	1601
TOTAL	1908	1121	2804	22,003	16,441	12,745

The maxi list (containing many more, but narrower disease categories, than the wide list) produced an estimate, 40 % higher than the WHO model, of 2253 (95 % CI 632–2904) deaths, being 5.1 % of all deaths. IHD, CHF lung cancer and stroke accounting for 41 %, 18 %, 16 % and 14 % of all attributable deaths respectively (Table 4).

**Table 4:** Mortality attributable to ambient air pollution from PM2.5 (Israel 2015) *(MAXI list–Single Pollutant Models)*

PM2.5	Deaths	95 % Lcl	95 % Ucl	Discounted		
				PYLL	HALE losses	HALE losses
Diabetes	78	24	122	820	600	481
LBW	0	0	0	17	15	6
Lung cancer	349	199	478	5945	4598	3384
COPD	78	−8	157	834	614	492
IHD	914	696	1142	11,118	8447	6457
CHF	417	180	618	4313	3194	2474
Stroke	278	−362	703	3659	2759	2078
ALRI	116	68	172	1155	853	657
Asthma	22	15	30	305	227	166
TOTAL	2253	811	3424	28,167	21,307	16,195

**Table 5:** Deaths, hospital utilization and costs from PM2.5 (Israel 2015)

	Total	WHO	WIDE	MAXI
Deaths	44,354	1609	1908	2253
		3.6 %	4.3 %	5.1 %
Hospital days	5,172,000	183,276	348,039	591,014
		3.5 %	6.7 %	11.4 %
General Hospital Costs	$4,495,153,288	$167,941,477	$318,918,656	$541,563,409
As % of Gen Hosp costs		3.7 %	7.1 %	12.0 %
All Health Costs	$22,432,880,000	$541,213,353	$1,027,757,036	$1,745,258,843
As % of all health costs		2.4 %	4.6 %	7.8 %
Mortality Costs				
Friction costs	$69,686,591	$2,461,150	$2,680,943	$3,366,447
Total Costs	$22,502,566,591	$543,674,503	$1,030,437,979	$1,748,625,290
% of GNP	7.6 %	0.18 %	0.35 %	0.59 %
Value of Statistical Life (GNP per capita based)	$16,346,340,983	$583,629,920	$631,308,623	$796,589,039

Table 5 shows that PM2.5 pollution accounted for between 183,000–591,000 days in general hospitals, costing between $168 million–$592 million, 3.5–11.4 % of all general hospital costs. Total health costs from PM2.5 pollution were between $541 million - $1028 million accounting for between 2.4–4.6 % of health expenditures in Israel. Total costs from PM2.5 pollution (including premature burial costs) amounted to between $544 million–$1749 million or 0.18 %–0.59 % of GNP. Using a statistical value of life based on GNP per capita methodology would add between $584 million–$797 million to the morbidity costs of PM2.5 pollution.

## Discussion

In contrast to deaths which are clearly attributable to a given causality (such as automobile accidents, suicides, drowning), deaths due to air pollution and to personal behaviour, such as smoking, nutritional habits and physical exercise are harder to identify. Despite this difficulty, ambient particulate matter pollution has been implicated as a factor in many causes of death [8].

The range of mortality from our three estimates of between 1609–2253 deaths from PM2.5 alone is between four and five times that of road accident fatalities (although road fatalities have a higher PYLL due to the younger age of deceased persons) and between 10–16 times that of homicides in Israel [10]. Mortality attributable to PM2.5 is however lower than deaths from smoking [21], obesity [22] and sedentariness [23].

Our estimated deaths from PM2.5 are lower than the 2452 estimated by the WHO European region in 2010 [1] partly due to our model taking into account the fact that the southern desert region of the country has higher particulate levels but a far lower population density.

Particulate matter data in Israel are strongly impacted by synoptic phenomena such as the occurrence of “dust storms” from surrounding deserts. Our estimates were limited to pollution data from only 2015, when there was a below average incidence of such storms. Hence our overall estimates of mortality, hospitalizations and costs are more likely to be downwardly biased than if they were to have been based on multi-year pollution data.

Our estimates were based on the 52 non-roadside monitoring stations, which fall far short of the current infeasible goal of having monitoring stations in every neighbourhood or street. These stations are not distributed randomly in the urban space, but are located after careful thought, often in places of special interest (e.g. potential hot spots, town halls etc.). Thus, averaging PM concentrations over monitoring stations (for either a city or a region) does not necessarily give a very good estimate of the true population exposure. In addition, there might also be data quality issues that need to be assessed and corrected by air pollution experts. Nevertheless, we consider our estimates to be an acceptable pragmatic compromise for the purpose of an initial estimation of the mortality effects from particulates. We consider our estimation method to be preferable to estimates based on industrial and transport emission volumes, where wind direction and natural pollutant sources such as sand act as confounders.

We consider the methodology for exposure assessment used in this paper to be a valid and generally acceptable for the purpose of making a national estimate of mortality. However, future localized estimates could be based on improved methodologies utilizing spatial models of particulate matter based on integrating data from monitoring stations, meteorology, traffic and other inputs.

A major limitation of our estimates is that due to the lack of such studies in Israel, we employed, as an acceptable compromise, relative risk estimates from studies in countries where the PM2.5 is at a different exposure level. In the event of non-linearity between risk and exposure this would cause biased estimates. However, these biases were lessened by our exclusion of Asian based studies, which tended to have higher PM2.5 levels.

A further source of potential bias is that the sources and hence composition of PM2.5 and subsequent composition-specific relative risks [24, 25] in international studies are different from that in Israel. While relying on meta analyses of risks might reduce any difference with Israel, an overall bias cannot be ruled out.

It should be borne in mind that our estimates only relate to one pollutant, particulate matter. A companion article will estimate the mortality attributable to two other air pollutants (Ozone and Nitrogen Dioxide). Due large negative and smaller positive correlations with particulate matter levels respectively, a simple addition of all three individual pollutant models will overestimate the total deaths attributable to ambient air pollution. Therefore adjustments will be made to the estimated total deaths by means of combining data from three studies [26–28] that have reported results of multi-pollution models (i.e.: that adjusted for the other two pollutants).

The WHO estimates, have a great advantage in that they allow for uniform comparisons with other countries, and that their relative risk information for IHD and Stroke was age-specific. However their disadvantage is that their RR were based on information that was available three years ago in 2013.

Our WIDE and MAXI lists incorporated data from studies on Diabetes, which had a significant RR. However, it could be considered contentious that we included categories whose RR were marginally significant (COPD, LBW) or not significant (Strokes), although Strokes were considered significant in the WHO model. The inclusion of LBW did not affect the WIDE estimates magnitude, since LBW contributed close to zero attributable deaths. However the inclusion of COPD and Strokes (in addition to LBW) in the MAXI list added 356 [95 % CI, −370, +860] deaths.

The mortality, morbidity (between 3.5 %–11.4 % of general hospital days) and monetary burden (between $544–$1748 million annually) of diseases attributable to air pollution in Israel is of sufficient magnitude to warrant the consideration and prioritisation of technological interventions that are available to reduce air pollution from industrial and vehicular sources.

While some interventions will be on a national scale (eg: limits on vehicle emissions), others might be aimed at local hot spots of high industry or vehicular pollution where a significantly large population is being exposed. Thus further analysis of our data (at pollution station level) will be required to identify and prioritise high risk localities and search for possible supplementary interventions (to national level interventions).

The data in this study provides a basis of mortality, DALY and health costs that can form the basis of any future cost-utility analyses of interventions (with proven efficacy) to reduce the burden of disease from man-made sources of particulate matter pollution. Interventions will have the potential not only to reduce mortality (and morbidity) but also to generate reductions in attributable health service costs that account for between 2.4 %–7.8 % of all health expenditures in Israel.

In the UK in 2005 [1, 29], road transport accounted for around 40 % of premature deaths from APMP, other transport (20 %), power generation (20 %) and other sectors (20 %). As long as twenty years ago, a considerable number of deaths from particulate matter in Tel-Aviv, Israel were shown to be attributable to diesel fuels [30]. Ways have been suggested to almost eradicate reduce these emissions and hence their related mortality and morbidity [31] by increasing the use of catalytic converters and moving over to hybrid, electrical and LPG powered vehicles–especially trucks and buses.

Large desert areas account for the fact that the Middle East is the region with the highest percentage of PM2.5 pollutants from natural sources [32], being around 52 % compared with 42 % Japan, 22 % Africa, 21 %, India, 17 % China, 10 % USA and 5 % Western Europe. So the potential for decreasing the percentage of particulate mass concentrations (used in this paper) through technological improvements is lower in the Middle East than in other regions (both developed and developing).

The effect of surrounding deserts on air Pollutant levels in Israel was described almost a decade ago [33]. A natural experimental study on the Day of Atonement from 2000–2008, when nearly all industry and vehicular travel ceases, based on four stations in three cities, reported a reduction in particulate concentrations ranging from 11.4 %–21.7 % [34]. However, a similar study over a longer period (1998–2012) estimated a 74 % contribution by natural sources to PM2.5 pollution [35].

Assuming 74 % of particulate pollution comes from natural sources in Israel, means that for every 10 % relative decrease in man-made PM2.5 attained through the implementation of intervention strategies [36], between 42–59 lives will be saved each year, (in addition to between $14 million and $21 million in resource costs).

## Conclusions

The considerable mortality and morbidity burden attributable to ambient particulate matter pollution, cries out for the establishment of an inter-ministerial plan to identify and implement those intervention strategies that are cost-effective, in order to decrease the considerable burden of mortality and morbidity, in both human and monetary terms, from ambient air pollution in Israel.

## Additional file

Additional file 1:
**Appendix I.** Studies contained in meta-analyses of RR due to 10 ug/m3 changes in PM2.5. (DOC 192 kb)

## Abbreviations

AAP: Ambient air pollution
ALRI: Acute Lower Respiratory tract Infection
APMP: Ambient Particular Matter Pollution
COPD: Chronic Obstructive Pulmonary Disease
DALY: Disability adjusted life year
GNP: Gross national product
HALE: Healthy Adjusted Life Expectancy
IHD: Ischemic Heart Disease
LBW: Low Birth Weight
LC: Lung cancer
PAF: Population Attributable Fraction
PM10: Particulate Matter Particles less than 10 micrometers in diameter
PM2.5: Particulate Matter Particles less than 2.5 micrometers in diameter
PYLL: Potential years of Life Lost
RR: Relative risk
UK: United Kingdom of Great Britain and Northern Ireland
WHO: World Health Organization

## References

[1] World Health Organization Regional Office for Europe, OECD. Economic cost of the health impact of air pollution in Europe: Clean air, health and wealth. Copenhagen: WHO Regional Office for Europe; 2015.

[2] World Health Organization. Burden of disease from household air pollution for 2012. Summary of results. World Health Organization: Geneva. http://www.who.int/phe/health_topics/outdoorair/databases/FINAL_HAP_AAP_BoD_24March2014.pdf. Accessed 16 Oct 2016.

[3] Lopez AD, Rodgers A, Vander Hoorn S, Murray CJ (2002). Comparative Risk Assessment Collaborating Group. Selected major risk factors and global and regional burden of disease. Lancet.

[4] Ezzati M, Hoorn SV, Lopez AD, Danaei G, Rodgers A, Mathers CD, Murray CJL, Lopez AD, Mathers CD, Ezzati M, Jamison DT, Murray CJL (2006). Comparative Quantification of Mortality and Burden of Disease Attributable to Selected Risk Factors. Global Burden of Disease and Risk Factors.

[5] Lim SS, Vos T, Flaxman AD, Danaei G, Shibuya K, Adair-Rohani H (2012). A comparative risk assessment of burden of disease and injury attributable to 67 risk factors and risk factor clusters in 21 regions, 1990–2010: a systematic analysis for the Global Burden of Disease Study 2010. Lancet.

[6] Smith KR, Bruce N, Balakrishnan K, Adair-Rohani H, Balmes J, Chafe Z (2014). Millions dead: how do we know and what does it mean? Methods used in the comparative risk assessment of household air pollution. Annu Rev Public Health.

[7] Bakar N, Rosenthal G, Gabai N. Estimate of external costs due to air-pollution from transport and industry in Israel. Department of Environmental and Social Sciences. Tel-Hai Academic College: Tel-Hai Acedemic College; 2012. In Hebrew.

[8] Monthly Environmental Quality Data, Ministry of Environmental Protection. http://www.svivaaqm.net/Default.rtl.aspx. Accessed 16 Oct 2016.

[9] Central Bureau of Statistics, Monthly Bulletin of Statistics, November 2015, Jerusalem 2015. http://www.cbs.gov.il/webpub/pub/text_page_eng.html?publ=93&CYear=2015&CMonth=11. Accessed 16 Oct 2016.

[10] Central Bureau of Statistics, Statistical Annual of Israel No 66 2015, Table 27.9 Jerusalem 2015. http://www.cbs.gov.il/reader/shnaton/templ_shnaton_e.html?num_tab=st27_09&CYear=2015. Accessed 16 Oct 2016.

[11] Burnett RT, Pope A, Ezzati M, Olives C, Lim SS, Mehta S, et al. An integrated risk function for estimating the global burden of disease attributable to ambient fine particulate matter exposure. Environ Health Perspect. 2014;122:397–403. http://dx.doi.org/10.1289/ehp.1307049. Accessed 16 Oct 2016. 10.1289/ehp.1307049 PMC3984213 24518036

[12] Ostro B. Outdoor air pollution: assessing the environmental burden of disease at national and local levels. Geneva: World Health Organization; 2004. WHO. Environmental Burden of Disease Series. No. 5. http://www.who.int/quantifying_ehimpacts/publications/ebd5/en/. Accessed 16 Oct 2016.

[13] Wang B, Xu D, Jing Z, Liu D, Yan S, Wang Y (2014). Effect of long-term exposure to air pollution on type 2 diabetes meelitus risk: a systemic review and meta-analysis of cohort studies. Eur J Endocrinol.

[14] Jacquemin B, Siroux V, Sanchez M, Carsin A-e, Shilkowski T, Adam M, et al. Ambient Air Pollution and Adult Asthma Incidence in Six European Cohorts (ESCAPE). Environ Health Perspect. 2015;123:613–21. http://ehp.niehs.nih.gov/1408206/. Accessed 16 Oct 2016. 10.1289/ehp.1408206 PMC4455584 25712593

[15] Zheng X-Y, Ding H, Jiang L-N, Chen S-W, Zheng J-P, Qui M, et al. Association between Air Pollutants and astma emergency Room Visits and Hospital admissions in Time series studies: A Systematic Review and Meta-Analysis. PLoS ONE. 10(9):e0138146. doi:10.1371/journal.pone.0138146. 10.1371/journal.pone.0138146 PMC4575194 26382947

[16] Pedersen M, Giorgis-Allemand L, Bernard C, Aguilera I, Andersen AM, Ballester F, et al. Ambient air pollution and low birthweights: a European cohort study [ESCAPE].The Lancet Respiratory Medicine 2013:1; 695–704. http://www.thelancet.com/pdfs/journals/lanres/PIIS2213-2600(13)70192-9.pdf. Accessed Oct 16 2016. 10.1016/S2213-2600(13)70192-9 24429273

[17] Kiomourtzoglou M-A, Schwartz JD, Weisskopf MG, Melly SJ, Wang Y, Dominici F, et al. Long-term PM2.5 Exposure and Neurological Hospital admissions in Northeastern United States. Environ Health Perspect. 2016;124:23–9. http://ehp.niehs.nih.gov/wp-content/uploads/124/1/ehp.1408973.alt.pdf. Accessed Oct 16 2016. 10.1289/ehp.1408973 PMC4710596 25978701

[18] Brookmeyer R, Evans A, Hebert L, Langa M, Heeringa G, Plassman L (2011). National estimates of the prevalence of Alzheimer’s disease in the United States. Alzheimers Dement.

[19] Faustini A, Rapp R, Forastiere F (2014). Nitrogen dioxide and mortality: review and meta-analysis of long-term studies. Eur Respir J.

[20] Ministry of Health. Ambulatory and Hospitalization Price Data for 2015. http://www.health.gov.il/subjects/finance/taarifon/pages/pricelist.aspx. Accessed 16 Oct 2016.

[21] Ginsberg G, Geva H (2014). The burden of smoking in Israel–attributable mortality and costs (2014). Isr J Health Policy Res.

[22] Ginsberg G, Rosen B, Rosenberg E. Cost-Utility Analysis Cost-Utility Analyses of Interventions to Prevent and Treat Obesity in Israel. RR-550-10. Brookdale-Smokler Center for health policy research. http://brookdale.jdc.org.il/?CategoryID=192&ArticleID=211. Accessed 16 Oct 2016.

[23] Ginsberg G, Rosen B, Rosenberg E. Cost-Utility Analysis Cost-Utility Analyses of Interventions to Increase Physical Activity in Israeli adults. RR-565-11, Brookdale-Smokler Center for health policy research. http://brookdale.jdc.org.il/?CategoryID=192&ArticleID=257. Accessed 16 Oct 2016.

[24] Wang M, Beelen R, Stafoggia M, Raaschou-Nielsen O, Andersen ZJ, Hoffman B (2014). Long-term exposure to elemental constituents of particulate matter and cardiovascular mortality in 19 European cohorts: Results from the ESCAPE and TRANSPHORM projects. Environ Int.

[25] Pedersen M, Gehring U, Beelen R, Wang M, Giorgis-Allmand L, Nybo A-M (2016). Elemental Constituents of Particulate Matter and newborn’s Size in Eight European Cohorts. Environ Health Perspect.

[26] Jerrett M, Burnett RT, Beckerman BS (2013). Spatial analysis of air pollution and mortality in California. Am J Respir Crit Care Med.

[27] Crouse DL, Petera PA, Hystad P, Brook JR, van Donkelaar A, Randall V (2015). Ambient PM2.5, O3, and NO2 Exposures and Associations with Mortality over 16 Years of Follow-up in the Canadian Census Health and Environment Cohort [CanCHEC]. Environ Health Perspect.

[28] Turner MC, Jerrett M, Pope III CA, Krewski D, Gatspur SM, Diver WR, et al. Long-Term Ozone Exposure and Mortality in a Large prospective Study. In press. Am J Respir Crit Care Med. doi:10.1164/rccm.201508-1633OC. Posted online on 17 Dec 2015. 10.1164/rccm.201508-1633OC PMC4872664 26680605

[29] Yim SHL, Barrett SRH (2012). Public health impacts of consumption emissions in the United Kingdom. Environ Sci Technol.

[30] Ginsberg GM, Seeri A, Fletcher E, Koutik D, Keresente E, Shemer Y (1998). Mortality and morbidity from vehicular emissions in Tel-Aviv. World Transp Policy Prac.

[31] Ginsberg GM, Seeri A, Fletcher E, Tene M, Karsente E, Shemer Y (1998). Mortality reductions as a result of changing to alternative powered vehicles in Tel-Aviv-Jafo. World Transp Policy Prac.

[32] Karagulian F, Belis C, Dor C, Pruss-Ustun A, Bonjour S, Adair-Rohani H (2015). Contributions to cities’ ambient particulate matter [PM]: A systematic review of local source contributions at global level. Atmos Environ.

[33] Erel Y, Dayan U, Rabi R, Rudich Y, Stein M (2006). Trans-boundary transport of pollutants by atmospheric mineral dust. Environ Sci Technol.

[34] Dayan U, Erel Y, Shpund J, Kordova L, Wanger A, Schauer JJ (2011). The impact of local sources and meteorological factors on nitrogen oxide and particulate matter concentrations: A case study of the Day of Atonement in Israel. Atmos Environ.

[35] Levy I. A national day with near zero emissions and its effect on primary and secondary pollutants. Atmospheric Environment. 2013:77;202–12.

[36] World Health Organization. Reducing Global Health Risks through mitigation of Short-Lived Climate Pollutants. Scoping Report for Policy-makers. Scovronick N, editor. Switzerland; 2015. ISBN: 978 92 4 156508 0.

