# Supplementary material for: Mortality, hospital days and expenditures attributable to ambient air pollution from particulate matter in Israel
Source: Isr J Health Policy Res. 2016 Nov 15;5:51. doi: 10.1186/s13584-016-0110-7 (PMC5109840; doi:10.1186/s13584-016-0110-7)
Supplement: Additional file 1: — Appendix I. Studies contained in meta-analyses of RR due to 10 ug/m3 changes in PM2.5. (DOC 192 kb) [file 13584_2016_110_MOESM1_ESM.doc]

| **Appendix I : Studies contained in meta-analyses of RR due to 10 ug/m3 changes in PM2.5** | | | | | | |
| --- | --- | --- | --- | --- | --- | --- |
|  |  |  |  |  |  |  |
|  |  |  |  | **Lower** | **Upper** | **Weight** |
|  |  | **Ref** | **RR** | **95% CL** | **95% CL** |  |
|  |  |  |  |  |  |  |
| **ALRI** |  |  |  |  |  |  |
| Burnett (WHO) | 2014 | 1 | **1.10** | **1.06** | **1.12** | **100%** |
|  |  |  |  |  |  |  |
| **ALZHEIMERS** |  |  |  |  |  |  |
| Kioumourtzoglou | 2016 | 2 | 3.00 | 2.40 | 3.70 | **100%** |
|  |  |  |  |  |  |  |
| **ASTHMA** |  |  |  |  |  |  |
| Jacquemin | 2015 | 3 | 1.08 | 0.76 | 1.46 | 0.05% |
| Zheng | 2015 | 4 | 1.023 | 1.015 | 1.031 | 99.95% |
|  |  |  | **1.02** | **1.01** | **1.03** | **100%** |
| **CHF** |  |  |  |  |  |  |
| Atkinson | 2013 | 5 | 1.15 | 1.02 | 1.27 | 21.9% |
| Pope | 2015 | 6 | 1.11 | 1.05 | 1.18 | 78.1% |
|  |  |  | **1.12** | **1.04** | **1.20** | **100%** |
|  |  |  |  |  |  |  |
| **COPD** |  |  |  |  |  |  |
| Burnett (WHO) | 2014 | 1 | 1.05 | 1.02 | 1.07 | 60.3% |
| Chan | 2015 | 7 | 1.00 | 0.96 | 1.04 | 26.9% |
| Crouse | 2015 | 8 | 0.98 | 0.93 | 1.13 | 7.4% |
| Hart | 2011 | 9 | 1.09 | 0.83 | 1.39 | 0.4% |
| Schikowski | 2014 | 10 | 1.06 | 0.73 | 1.53 | 0.2% |
| Turner | 2015 | 11 | 1.10 | 1.02 | 1.19 | 4.7% |
|  |  |  | **1.03** | **1.00** | **1.07** | **100%** |
| **DEMENTIA** |  |  |  |  |  |  |
| Kioumourtzoglou | **2016** | **2** | **1.16** | **1.10** | **1.22** | **100%** |
|  |  |  |  |  |  |  |
| **DIABETES** |  |  |  |  |  |  |
| Brook | 2013 | 12 | 1.49 | 1.37 | 1.62 | 1.1% |
| Chen | 2013 | 13 | 1.11 | 1.02 | 1.21 | 1.9% |
| Coogan | 2012 | 14 | 1.15 | 0.51 | 2.58 | 0.0% |
| Crouse | 2015 | 8 | 1.30 | 1.23 | 1.37 | 3.1% |
| Goldberg | 2001 | 15 | 1.08 | 1.02 | 1.13 | 5.6% |
| Goldberg | 2013 | 16 | 1.03 | 1.00 | 1.05 | 30.0% |
| Kramer | 2010 | 17 | 1.02 | 0.97 | 1.10 | 4.3% |
| Ostro | 2006 | 18 | 1.02 | 1.01 | 1.04 | 51.2% |
| Pope | 2015 | 6 | 1.13 | 1.02 | 1.26 | 1.2% |
| Puett | 2011 | 19 | 1.08 | 0.90 | 1.25 | 0.5% |
| Turner | 2015 | 11 | 1.07 | 0.96 | 1.20 | 1.2% |
|  |  |  | **1.05** | **1.01** | **1.08** | **100%** |
|  |  |  |  |  |  |  |
| **IHD** |  |  |  |  |  |  |
| Beelen | 2014 | 20 | 0.96 | 0.48 | 1.60 | 0.1% |
| Burnett (WHO) | 2014 | 1 | 1.07 | 1.05 | 1.11 | 32.8% |
| Cesaroni | 2013 | 21 | 1.10 | 1.06 | 1.13 | 18.2% |
| Crouse | 2015 | 8 | 1.17 | 1.15 | 1.20 | 31.6% |
| Gan | 2011 | 22 | 1.06 | 0.94 | 1.32 | 0.8% |
| Hart | 2011 | 9 | 1.02 | 0.91 | 1.15 | 1.5% |
| Hart | 2015 | 23 | 0.99 | 0.88 | 1.10 | 1.7% |
| Pope | 2015 | 6 | 1.14 | 1.10 | 1.18 | 13.1% |
| Villeneuve | 2015 | 24 | 1.34 | 1.09 | 1.66 | 0.3% |
|  |  |  | **1.11** | **1.08** | **1.15** | **100%** |
|  |  |  |  |  |  |  |
| **LBW** |  |  |  |  |  |  |
| Davand | 2013 | 25 | 1.04 | 0.99 | 1.09 | 15.6% |
| Huynh | 2006 | 26 | 1.15 | 1.07 | 1.24 | 5.5% |
| Madsen | 2010 | 27 | 1.04 | 1.02 | 1.06 | 81.3% |
| Pedersen | 2013 | 28 | 1.42 | 1.12 | 1.76 | 0.4% |
| Salam | 2005 | 29 | 1.12 | 0.76 | 1.16 | 12.0% |
| Sapkota | 2012 | 30 | 1.09 | 0.90 | 1.32 | 0.9% |
|  |  |  | **1.06** | **0.99** | **1.09** | **100%** |
|  |  |  |  |  |  |  |
| **LUNG CANCER** |  |  |  |  |  |  |
|  |  |  |  |  |  |  |
| Boldo | 2006 | 31 | 1.14 | 1.04 | 1.23 | 3.3% |
| Brunkeef | 2009 | 32 | 1.06 | 0.82 | 1.38 | 0.4% |
| Carey | 2013 | 33 | 1.21 | 0.95 | 1.47 | 0.4% |
| Cesaroni | 2013 | 21 | 1.05 | 1.01 | 1.10 | 15.2% |
| Crouse | 2015 | 8 | 1.06 | 1.03 | 1.10 | 22.8% |
| Fischer | 2015 | 34 | 1.19 | 1.15 | 1.22 | 28.4% |
| Hart | 2011 | 9 | 1.06 | 0.93 | 1.20 | 1.6% |
| Heinrich | 2013 | 35 | 1.88 | 1.24 | 2.81 | 0.1% |
| Hystad | 2013 | 36 | 1.29 | 0.95 | 1.76 | 0.2% |
| Krewski/Turner (a) | 2009/15 | 37/11 | 1.08 | 1.01 | 1.15 | 6.5% |
| Lepeule | 2012 | 38 | 1.37 | 1.07 | 1.75 | 0.3% |
| Lipsett | 2011 | 39 | 0.95 | 0.70 | 1.28 | 0.4% |
| McDonnell | 2000 | 40 | 1.39 | 0.79 | 2.46 | 0.1% |
| Naess | 2007 | 41 | 1.08 | 1.04 | 1.12 | 18.5% |
| Peutt | 2014 | 42 | 1.06 | 0.90 | 1.24 | 1.0% |
| Raachou | 2013 | 43 | 1.32 | 0.84 | 1.90 | 0.1% |
| Villeneuve | 2015 | 24 | 0.97 | 0.80 | 1.18 | 0.8% |
|  |  |  | **1.11** | **1.05** | **1.16** | **100%** |
| **PARKINSONS** |  |  |  |  |  |  |
| Kioumourtzoglou | 2016 | 2 | **1.88** | **1.44** | **2.40** | **100%** |
|  |  |  |  |  |  |  |
| **RESPIRATORY** |  |  |  |  |  |  |
| Carey | 2013 | 35 | 1.63 | 1.42 | 1.84 | 0.4% |
| Crouse | 2015 | 12 | 0.95 | 0.91 | 0.98 | 13.6% |
| Dimakopolou | 2014 | 44 | 0.78 | 0.32 | 1.24 | 0.1% |
| Faustini | 2014 | 45 | 1.05 | 1.01 | 1.09 | 10.3% |
| Fischer | 2015 | 8 | 1.09 | 1.07 | 1.12 | 30.2% |
| Hao | 2015 | 46 | 1.14 | 0.98 | 1.28 | 0.8% |
| Hart | 2011 | 13 | 1.10 | 0.90 | 1.34 | 0.4% |
| Heinrich | 2013 | 36 | 0.97 | 0.71 | 1.39 | 0.2% |
| Jerrett | 2009 | 47 | 1.03 | 0.96 | 1.11 | 3.0% |
| Laden | 2006 | 48 | 1.08 | 0.79 | 1.49 | 0.2% |
| Lipsett | 2011 | 40 | 1.21 | 0.97 | 1.52 | 0.3% |
| Ostro | 2010 | 49 | 1.03 | 0.80 | 1.34 | 0.3% |
| Perez | 2015 | 50 | 1.02 | 0.99 | 1.04 | 26.9% |
| Pope | 2004 | 51 | 0.92 | 0.86 | 0.98 | 5.2% |
| Thurston | 2015 | 52 | 1.05 | 0.98 | 1.13 | 3.3% |
| Turner | 2015 | 9 | 1.16 | 1.10 | 1.23 | 4.5% |
| Villeneuve | 2015 | 24 | 0.82 | 0.61 | 1.11 | 0.3% |
|  |  |  | **1.04** | **1.00** | **1.08** | **100%** |
| **STROKE** |  |  |  |  |  |  |
| Hart | 2015 | 23 | 1.03 | 0.92 | 1.15 | 72.0% |
| Scheers | 2015 | 53 | 1.16 | 0.96 | 1.38 | 21.8% |
| Villeneuve | 2015 | 24 | 1.31 | 0.97 | 1.78 | 6.2% |
|  |  |  | **1.08** | **0.93** | **1.24** | **100%** |

|  |  |  |  |  |  |  |
| --- | --- | --- | --- | --- | --- | --- |

**References for Appendix I**

1.Burnett RT, Pope A, Ezzati M, Olives C, Lim SS, Mehta S, et al. An integrated risk function for estimating the global burden of disease attributable to ambient fine particulate matter exposure. Environ Health Perspect 2014;122;4;397-403. <http://dx.doi.org/10.1289/ehp.1307049> Accessed Jan 6th 2016. 17.

2. Kiomourtzoglou M-A, Schwartz JD, Weisskopf MG, Melly SJ, Wang Y, Dominici F. et al. et al. Long-term PM2.5 Exposure and Neurological Hospital admissions in Northeastern United States. Environ Health Perspect Jan 2016;124: 23-9. http;//dx.do.org/ 10.1289/ehp.1408973.

3. Jacquemin B, Siroux V, Sanchez M. Carsin A-e, Shilkowski T, Adam M. et al. Ambient Air Pollution and Adult Asthma Incidence in Six European Cohorts (ESCAPE). Environ Health Perspect June 2015;123: 613-21. <http://dx.do.org/10.1289/ehp.1408206>

4.Zheng X-Y, Ding H, Jiang L-N, Chen S-W, Zheng J-P, Qui M. et al. association between Air Pollutants and asthma emergency Room Visits and Hospital admissions in Time series studies: A Systematic Review and Meta-Analysis. PLoS ONE 1099): e0138146. Doi: 10;1371/journal.pone.0138146

5. Atkinson RW, Carey IM, Kent AJ, van Staa TP, Anderson HR, Cooka DG. Long-Term Exposure to Outdoor Air Pollution and Incidence of Cardiovascular Diseases. Epidemiology 2013; 24: 44–53

6. Pope CA III, Turner MC, Burnett RT et al. Relationships Between Fine Particulate Air Pollution, Cardiometabolic Disorders, and Cardiovascular Mortality. Circ Res. 2015;116:108-115. DOI: 10.1161/CIRCRESAHA.116.305060.

7.Chan MPL, Weinhold RS, Thomas R, Gohlke JM, Portier CJ. Environmental predictors of US County Mortality Patterns on a National Basis. PLOS ONE 2015; 10 [12]: e0137832 | DOI:10.1371/journal.pone.0137832.

8.Crouse DL, Petera PA, Hystad P, Brook JR, van Donkelaar A, Randall V et al. Ambient PM2.5, O3, and NO2 Exposures and Associations with Mortality over 16 Years of Follow-up in the Canadian Census Health and Environment Cohort [CanCHEC]. Environmental Health Perspectives 2015;123;11: 1180-1186.

9. Hart JE, Garshick E, Dockery DW, Smith TJ, Ryan L, Laden F. Long-Term Ambient Multipollutant Exposures and Mortality. Am J Resp Crit care Med 2011;183;73-78.

10.Schikowski T, Adam M, Marcon A et al. Association of ambient air pollution with the prevalence and incidence of COPD. Eur Respir J 2014; 44: 614–626 DOI: 10.1183/09031936.00132213

11.Turner MC, Jerrett M, Pope III CA, Krewski D, Gatspur SM, Diver WR et al. long-Term Ozone Exposure and Mortality in a Large prospective Study. In press: [American Journal of Respiratory and Critical Care Medicine](http://www.atsjournals.org/loi/ajrccm)10.1164/rccm.201508-1633OC. Posted online on 17 Dec 2015.

12.Brook RD, Cakmak S, Turner MC et al. Long-Term Fine Particulate Matter Exposure and Mortality from Diabetes in Canada Diabetes Care 2013; 36: 3313–3320.

13. Chen H, Burnett RT, Kwong JC et al. Risk of Incident Diabetes in Relation to Long-term Exposure to Fine Particulate Matter in Ontario, Canada. Environ Health Perspect 2013;121:804-810]; DOI:10.1289/ehp.1205958

14. Coogan PF, White LF, Jerrett M, Brook RD, Su JG, Seto E, et al. Air pollution and incidence of hypertension and diabetes mellitus in black women living in Los Angeles: clinical perspective. Circulation 2012: 125:767–772.

15. Goldberg MS, Burnett RT, Brook J, Ballar III JC, Valois M-F, Vincent R. Associations between daily cause-specific mortality and concentrations of Ground- level Ozone in Montreal, Quebec. Am J Epidemiol 2001;154:317-326 doi: 10/1093/aje/154.9.817

16. Goldberg MS, Burnett RT, Stieb DM, et al. [Associations between ambient air pollution and daily mortality among elderly persons in Montreal, Quebec.](http://www.ncbi.nlm.nih.gov/pubmed/23872247) Sci Total Environ. 2013;463-464:931-42. doi: 10.1016/j.scitotenv.2013.06.095. Epub 2013 Jul 19.

17. Krämer U, Herder C, Sugiri D, Strassburger K, Schikowski T, Ranft U, et al. Traffic-related air pollution and incident type 2 diabetes: results from the SALIA Cohort Study. Environ Health Perspect 2010;118:1273–1279.

18. Ostro B, Broadwin R, Green S, Feng W-Y, Lipsett M. Fine Particulate Air Pollution and Mortality in Nine California Counties: Results from CALFINE. Environ Health Perspect 2006;114:29–33. doi:10.1289/ehp.8335 available via <http://dx.doi.org/>

19.Puett RC, Hart JE, Schwartz J, Hu FB, Liese AD, Laden F. Are particulate matter exposures associated with risk of type 2 diabetes? Environ Health Perspect 2011; 119:384–389.

20.Beelen R, Stafoggia,M, Raaschou-Nielsen O.Long-term Exposure to Air Pollution and Cardiovascular Mortality.An Analysis of 22 European Cohorts. Epidemiology 2014;25: 368–378.

21. Cesaroni G, Badaloni C, Gariazzo Cl. Long-term exposure to urban air pollution and mortality in a cohort of more than a million adults in Rome. Environ Health Perspect 2013;121:324–331.

22. Gan WQ, Koehoorn M, Davies HW, Demers PA, Tamburic L, Brauer M. Long-Term Exposure to Traffic-Related Air Pollution and the Risk of Coronary Heart Disease Hospitalization and Mortality. Environ Health Perspect 119:501–507 [2011]. doi:10.1289/ehp.1002511

23. Hart JE, Puett RC, Rexrode KM, Albert CM, Laden F. Effect modification of long-term air pollution exposures and the risk of incident cardiovascular disease in US women. J Am Heart Assoc 2015;4:e002301 doi: 10.1161/JAHA.115.002301

24. Villeneuve PJ,. Weichenthal SA, Crouse D. et al. Long-term Exposure to Fine Particulate Matter Air Pollution and Mortality Among Canadian Women. Epidemiology 2015;26: 536–545.

25. Davand P, Parker J, Bell ML, et al. Maternal exposure to particulate air pollution and term birth weight; a multi-country evaluation of effect and heterogeneity. Environ Health Perspect 2013; 121: 267–373.

26. Huynh M. Relationship between air pollution and preterm birth in California. Pediatric and Perinatal Epidemiology 2006;20:454-461

27. Madsen C, Gehring U, Walker SE, Brunekreef B, Stigum H, Naess O, Nafstad P. Ambient air pollution exposure, residential mobility and term birth weight in Oslo, Norway. Environmental Research 2010;110: 363-371.

28. Pedersen M, Giorgis-Allemand L, Bernard C, et al. Ambient air pollution and low birthweight: a European cohort study [ESCAPE]. www.thelancet.com/respiratory Published online October 15, 2013 [http://dx.doi.org/10.1016/S2213-2600[13]70192-9](http://dx.doi.org/10.1016/S2213-2600(13)70192-9)

29. Salam MT, Millstein J, Li Y-F, Lurmann FW, Margolis HG, Gilliland FD.Birth Outcomes and Prenatal Exposure to Ozone, Carbon Monoxide, and Particulate Matter: Results from the Children’s Health Study. Environ Health Perspect 113:1638–1644 [2005]. doi:10.1289/ehp.8111

30. Sapkota A, Chelikowsky AP , Nachman KE, Cohen AJ, Ritz B. Exposure to particulate matter and adverse birth outcomes: a comprehensive review and meta-analysis. Air Qual Atmos Health 2012; 5: 369–381.

31. Boldo E, Medina S, LeTertre A, Hurley F, Mücke HG, Ballester F, Aguilera I, Eilstein D; Apheis Group. [Apheis: Health impact assessment of long-term exposure to PM[2.5] in 23 European cities.](http://www.ncbi.nlm.nih.gov/pubmed/16826453) Eur J Epidemiol. 2006;21:449-58. Epub 2006 Jul 7.

32. Brunekreef B, Beelen R, Hoek G, et al. Effects of long-term exposure to traffic related air pollution on respiratory and cardiovascular mortality in the Netherlands: the NLCS-AIR study. Res Rep Health Eff Inst 2009;139:5–71. discussion 73–89.

33. Carey IM, Atkinson RW, Kent AJ, van Staa T, Cook DG, Anderson HR. Mortality associations with long-term exposure to outdoor air pollution in a national English cohort. Am J Respir Crit Care Med 2013;187:1226–1233.

34. Fischer PH, Marra M, Ameling CB, et al. Air pollution and mortality in seven million adults: the Dutch Environmental Longitudinal Study [DUELS]. Environ Health Perspect 2015;123:697–704; http://dx.doi.org/10.1289/ehp.1408254

35. Heinrich J, Thiering E, Rzehak P, et al. Long-term exposure to NO2 and PM10 and all-cause and cause-specific mortality in a prospective cohort of women. Occup Environ Med 2013;70:179–186. doi:10.1136/oemed-2012-100876

36. Hystad P, Demers PA, Johnson KC, Carpiano RM, Brauer M. Long-term residential exposure to air pollution and lung cancer risk. Epidemiology 2013; 24[5]:762–772.

37. Krewski D, Jerrett M, Burnett RT et al. Extended follow-up and spatial analysis of the American Cancer Society study linking particulate air pollution and mortality. HEI Research Report 140, Health Effects Institute, Boston, MA. 2009; 140:5-114, discussion115-136.

38. Lepeule J, Laden F, Dockery D, Schwartz J. Chronic exposure to fine particles and mortality: an extended follow-up of the Harvard Six Cities study from 1974 to 2009. Environ Health Perspect 2012;120:965–970; doi:10.1289/ehp.1104660.

39. Lipsett MJ, Ostro BD, Reynolds P, et al. Long-term exposure to air pollution and cardiorespiratory disease in the California teachers study cohort. Am J Respir Crit Care Med. 2011;184:828–835.

40. McDonnell WF, Nishino-Ishikawa N, Petersen FF, Chen LH, Abbey DE. [Relationships of mortality with the fine and coarse fractions of long-term ambient PM10 concentrations in nonsmokers.](http://www.ncbi.nlm.nih.gov/pubmed/11051533) J Expo Anal Environ Epidemiol. 2000 Sep-Oct;10[5]:427-36.

41. Naess Ø, Nafstad P, Aamodt G, Claussen B, Rosland P. Relation between concentration of air pollution and cause-specific mortality: four-year exposures to nitrogen dioxide and particulate matter pollutants in 470 neighborhoods in Oslo, Norway. Am J Epidemiol 2007; 165:435–443.

42. Puett RC, Hart JE, Yanosky JD, Spiegelman D, Wang M, Fisher JA, Hong B, Laden F. Particulate matter air pollution exposure, distance to road, and incident lung cancer in the Nurses’ Health Study Cohort. Environ Health Perspect 2014;122:926–932; http://dx.doi. org/10.1289/ehp.1307490

43. Raaschou-Nielsen O, Andersen ZJ, Beelen R, Samoli E, Stafoggia M, Weinmayr G et al. Air pollution and lung cancer incidence in 17 European cohorts: prospective analyses from the European Study of Cohorts for Air Pollution Effects [ESCAPE]. Lancet Oncol 2013;14:813–822.

44. Dimakopoulou K, Samoli E, Beelen R, et al. Air pollution and nonmalignant respiratory mortality in 16 cohorts within the ESCAPE project. Am J Respir Crit

Care Med. 2014;189:684–696.

45. Faustini A, Rapp R, Forastiere F.Nitrogen dioxide and mortality: review and meta-analysis of long-term studies. Eur Respir J 2014; 44: 744–753 | DOI: 10.1183/09031936.00114713

46. Hao Y, Balluz L, Strosnider H, Wen XJ, Li C, Qualters JR. Ozone, Fine Particulate Matter, and Chronic Lower Respiratory Disease Mortality in the United States. Am J Respir Crit Care Med 2015; 192, 3, 337–341. doi: 10.1164/rccm.201410-1852OC

47. Jerrett M, Burnett RT, Pope CA III, et al. Long-term ozone exposure and mortality. N Engl J Med 2009; 360[11]:1085–1095.

48. Laden F, Schwartz J, Speizer FE, Dockery DW: Reduction in fine particulate Air pollution and mortality: extended follow-up of the Harvard Six cities study. Am J Respir Crit Care Med 2006, 173:667–672.

49. Ostro B, Lipsett M, Reynolds P, Goldberg D, Hertz A, Garcia C, Henderson KD, Bernstein L: Long-term exposure to constituents of fine particulate air pollution and mortality: results from the California teachers study. Environ Health Perspect 2010, 118:363–369.

50. Perez L, Grize L, Infanger D et al. associations of daily levels of PM10 and NO2 with emergency hospital admissions and mortality in Switzerland: Trends and missed prevention potential over the last decade. Environmental Research 2015; 140;554-561.

51. Pope CA III, Burnett RT, Thurston GD, et al. Cardiovascular mortality and long-term exposure to particulate air pollution: epidemiological evidence of general

pathophysiological pathways of disease. Circulation 2004; 109:71–77.

52. Thurston GD, Ahn J, Cromar KR, Shao Y, Reynolds HR, Jerrett M et al.

Ambient Particulate Matter Air Pollution Exposure and Mortality in the NIH-AARP Diet and Health Cohort Environ Health Perspect DOI:10.1289/ehp.1509676

53. Scheers H, Jacobs L, Casas L, Nemery B, Nawrot YS. Long-Term Exposure to Particulate Matter Air Pollution Is a Risk Factor for Stroke Meta-Analytical EvidenceStroke. 2015;46:3058-3066. DOI: 10.1161/STROKEAHA.115.009913.
